# Medical laser hair removal: a new rotational approach

**DOI:** 10.1007/s10103-025-04592-8

**Published:** 2025-08-19

**Authors:** Stefano Bacchini, Benedetta Mariani, Gian Marco Tomassini, Alberto Oddo, Roberto Cuomo, Ishith Seth, Warren Matthew Rozen, Luca Grimaldi, Alvaro Pacifici

**Affiliations:** 1 Department of Medicine, Surgery and Neuroscience - Plastic and Reconstructive Surgery Unit, University of Siena, 53100 Siena, Italy; 2 Department of Medicine and Surgery, University of Perugia, 06132 Perugia, Italy; 3 Clinica Laser Perugia, Perugia, Italy; 4 Department of Plastic Surgery, University of Melbourne, Melbourne, Australia

**Keywords:** Hair removal, Laser, Selective phototermolysis, Hypertrichosis, Hirsutism

## Abstract

Medical laser hair removal is a common treatment for both aesthetic and pathological conditions, permitting the reduction of dark and thick terminal hairs and increasing the number of vellus hairs. This study aims to present the clinical outcomes and safety of a new approach for hair removal, combining Nd: YAG (1064 nm), diode (808–810 nm), and Alexandrite (755 nm) lasers in a rotational regimen. From January 2022 to December 2023, 60 patients underwent a complete course of hair removal treatment, which included three sessions with Nd: YAG (1064 nm), diode (808–810 nm), and Alexandrite (755 nm) lasers in a rotational regimen. Follow-up visits were conducted three and six months after the last treatment session to assess the results. Across all treated body areas, the hair reduction at six months was 75.07%, with a significant reduction in hair thickness and an increase in vellus hairs. Additionally, the treatment was well-tolerated, with patients reporting only slight discomfort during laser irradiation, reflected by average pain scores of 2/4. No long-term adverse effects were observed, and transient side effects, such as erythema and perifollicular edema, resolved within days. This new rotational regimen for laser hair removal has proven to be effective, safe, and satisfactory for patients. The tailored approach enhances the treatment’s efficacy and ensures comprehensive targeting of various hair types and colors. This protocol promises a significant advancement in the field of laser hair removal, offering a reliable and patient-friendly solution for long-term hair reduction.

## Introduction

Hair reduction, sought for both aesthetic and pathological conditions such as hypertrichosis and hirsutism, is a frequent request among patients in dermatology clinics. Various laser wavelengths and intense pulsed light (IPL) systems are available, with these modalities representing the most enduring and effective options for hair reduction.

The most effective and commonly utilized laser types include Alexandrite (755 nm), diode lasers (808–810 nm), and Nd: YAG (1064 nm) lasers [1]. These modalities offer several levels of absorption with selective photothermolysis targeting melanin in the hair shaft and hair matrix [2, 3]. Melanin absorbs light energy, converting it into intense heat energy, which then diffuses and destroys the hair follicle, causing collateral damage to the stem cells located in the hair bulge [2]. This mechanism of action is reflected in the progressive reduction of dark, thick terminal hairs and a simultaneous increase in the number of vellus hairs, which are too small to be seen with the naked eye [4, 5]. More commonly, temporary hair loss occurs through the modification of the hair growth cycle and the induction of a kenogen-like state, in which the hair follicles are at rest, and no hair growth occurs [6].

The combination of various laser systems with different pigment-absorbing wavelengths enables the treatment of multiple depths and the ability to target various hair types and colors. The aim of this manuscript is to present and describe the clinical outcomes and safety of a new approach for hair removal, combining Nd: YAG (1064 nm), diode (808–810 nm), and Alexandrite (755 nm) lasers in a rotational regimen.

## Materials and methods

This prospective, open-label study was conducted at Clinica Laser Perugia and the Plastic Surgery Department of Siena, Italy, with Institutional Review Board (IRB) ethics approval obtained. The study adhered to the principles outlined in the Helsinki Declaration and was conducted from January 2022 to December 2023. Clinical trial number: not applicable. A total of 60 patients were included in the study, all of whom provided informed consent for the procedures and the publication of images and results.

Participants underwent a comprehensive hair removal treatment regimen consisting of three initial sessions with Nd: YAG (1064 nm), diode (808–810 nm), and Alexandrite (755 nm) lasers. Each session was performed with a different wavelenght in a temporally sequential manner. The three treatment sessions were scheduled at 4- to 6-week intervals. Subsequent sessions were performed as needed based on hair regrowth, continuing until the hair was reduced to fine, miniaturized vellus hair. Follow-up visits were conducted three and six months after the final treatment session to assess the outcomes.

For each Fitzpatrick skin type and corresponding terminal hair color, a specific wavelength laser was employed. In patients with skin types IV, V, and VI and dark hair, treatment commenced with Nd: YAG (1064 nm). After several sessions, the hair became thinner and lighter brown, at which point it was targeted with the diode laser (808–810 nm). This laser further transformed the hair, making it finer and blonder. Finally, the Alexandrite laser (755 nm), with its selective photothermolysis for blonde hair, was used. Conversely, the Alexandrite laser was the only treatment for patients with Fitzpatrick skin types I and II, aiming to achieve hair fleece in the kenogen phase.

The study included patients aged 18 to 60 years who underwent the rotational laser treatment approach. Treated body areas included: (i) arms, (ii) underarms, (iii) legs, (iv) bikini line, (v) chest, and (vi) back. Exclusion criteria were the presence of other dermatological conditions affecting the treatment area (e.g., dermatitis, malignant lesions, infection), or the use of systemic isotretinoin or anticoagulant drugs within the previous six months.

Sequential digital photographs using identical camera equipment were obtained at baseline, before each treatment session, and during follow-up visits. Dermatoscopic imaging of the treated area was assessed at three and six months post-treatment to measure treatment efficacy based on hair reduction percentage, hair density (N/cm²), and hair thickness (mm). Additionally, the percentage of vellus hair (diameter less than 40 μm) was calculated and reported. Adverse events occurring during treatment and up to the follow-up visit were recorded to evaluate the safety of the proposed treatment. Subject tolerability was assessed using a questionnaire and a four-point pain scale (1 = no discomfort; 2 = slight discomfort; 3 = moderate discomfort; 4 = severe discomfort).

## Result

The results obtained with the new rotational approach are summarized in Table 1.

**Table 1:** Hair reduction, hair density, hair thickness and Vellus hair before and after laser treatment

Variable	Assesment	Mean
Hair reduction (%)	At 6 months	75.07
Hairs density (N/cm2)	Before After	60 60
Hair thickness (µm)	Before After	250 40
Vellus hair (%)	Before After	20 90

Specifically, considering all treated body areas, the hair reduction percentage at six months post-treatment is 75.07%, accompanied by a significant reduction in hair thickness and an increase in the presence of vellus hair. However, the hair density remained unchanged before and after the treatment.

The data regarding the treatment’s safety were encouraging. Skin reactions observed during the laser sessions included erythema (99%) and perifollicular edema (98%). These side effects were transient and resolved within a few days for all patients. No permanent skin effects, such as scarring or pigmentary alterations, were observed during follow-ups or reported.

**Fig. 1:**
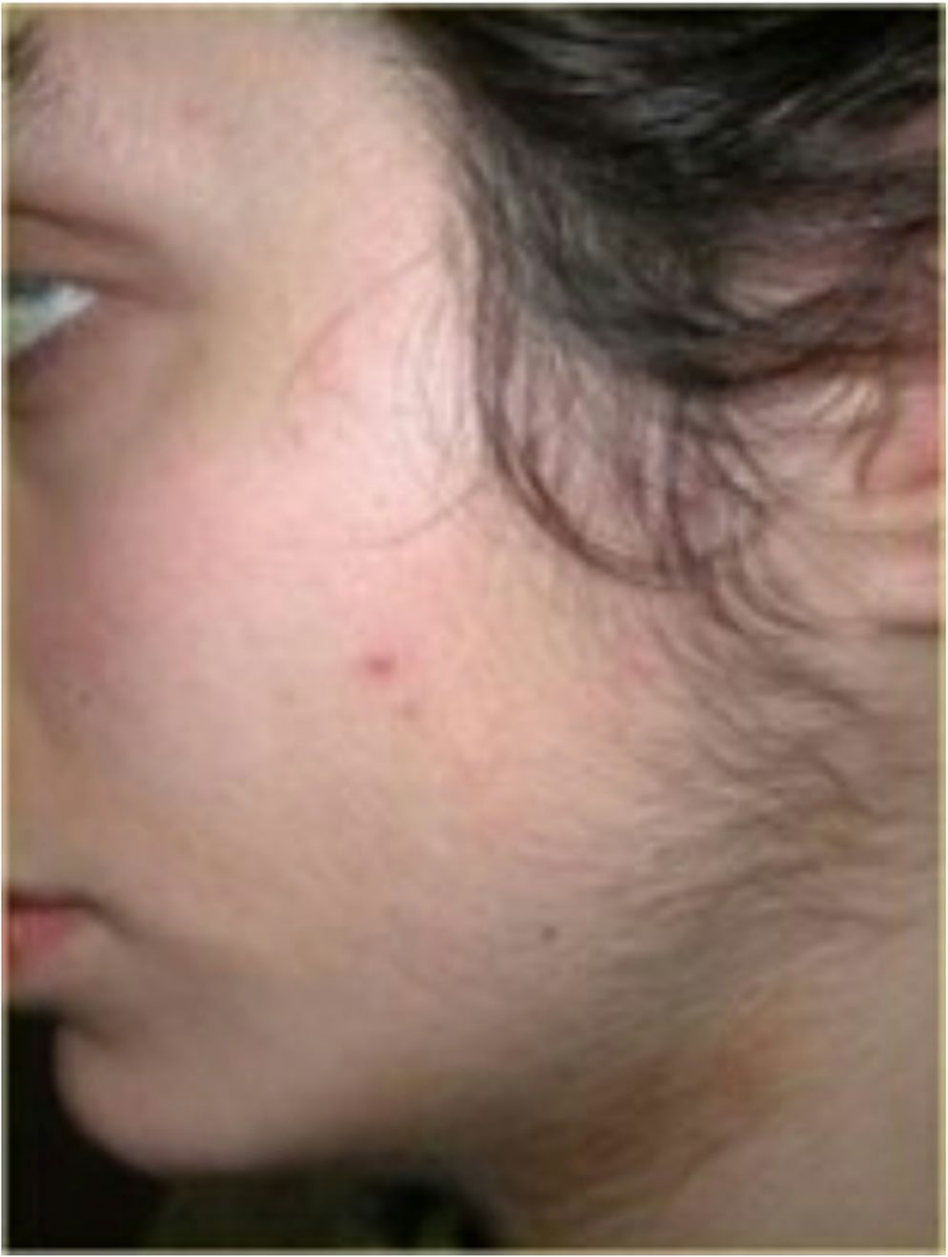
Laser hair removal of the beard. Before treatment

**Fig. 2:**
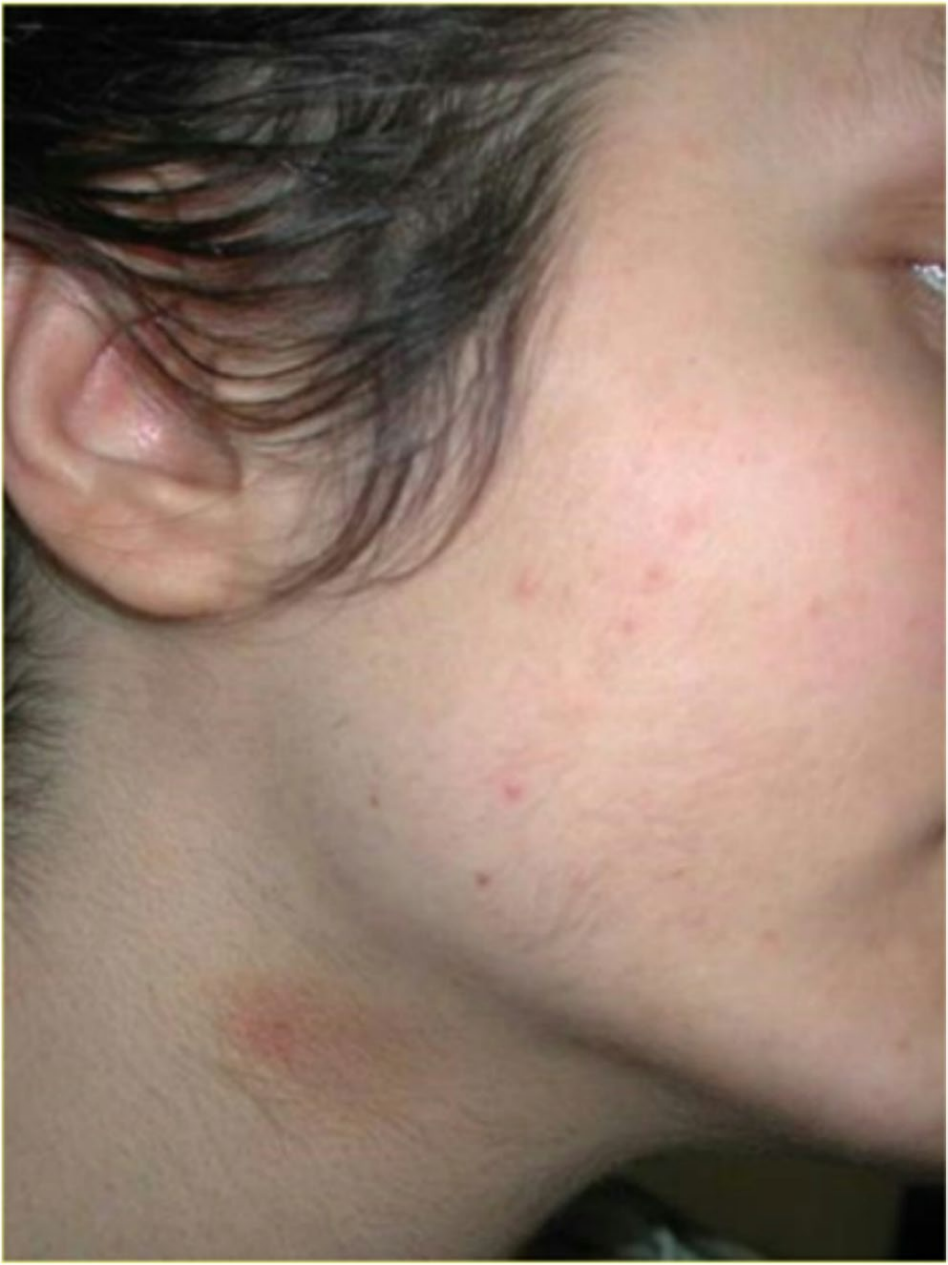
Laser hair removal of the beard. After 6 months of treatment

**Fig. 3:**
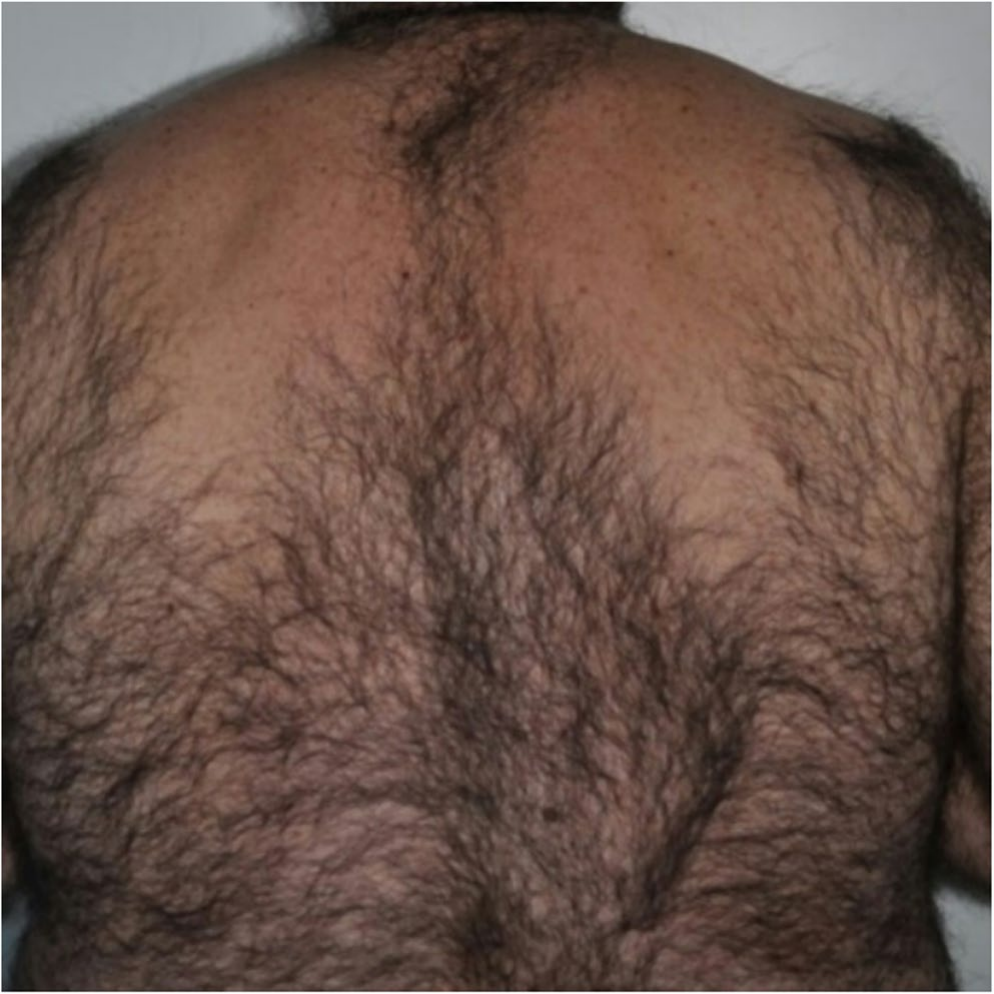
Laser hair removal of the back. Before treatment

**Fig. 4:**
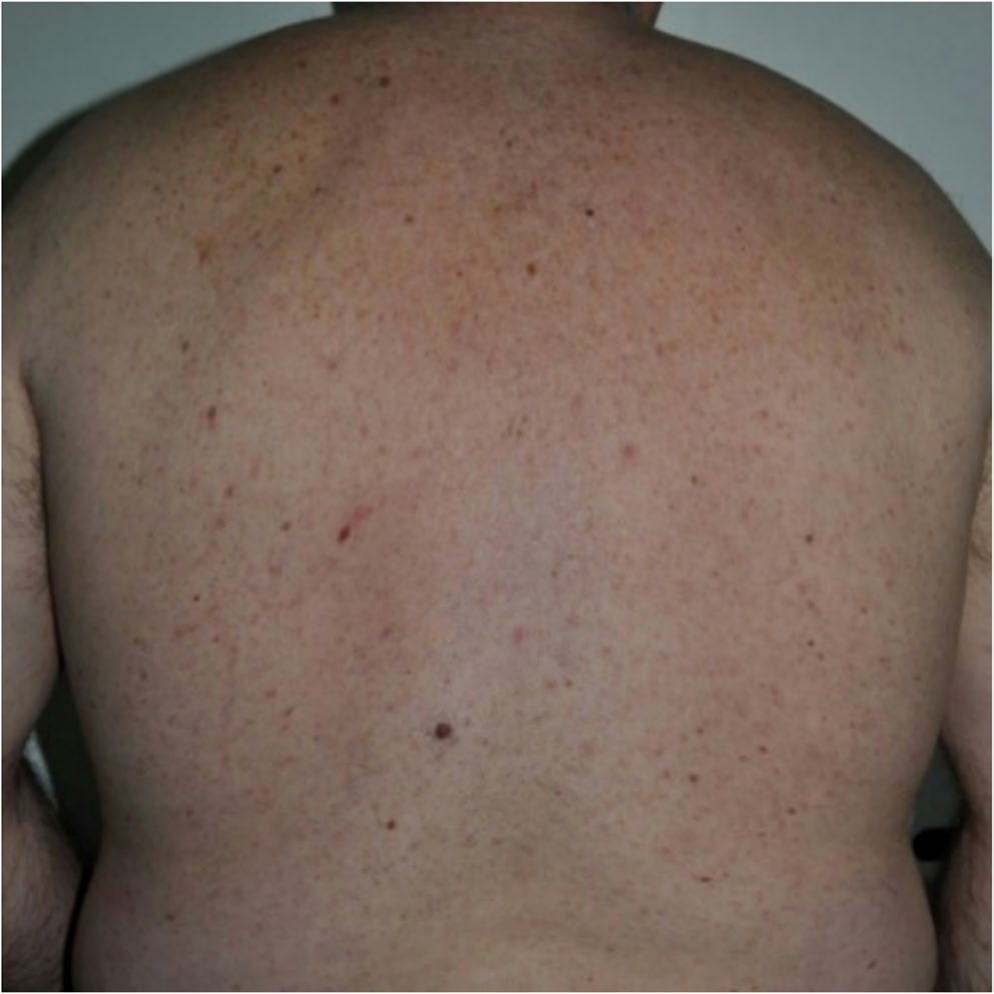
Laser hair removal of the back. After 6 months of treatment

According to subject questionnaires, only slight discomfort was reported during laser irradiation, with an average pain score of 2/4. Both the diode (808–810 nm) and Alexandrite (755 nm) lasers were well tolerated, with average pain scores of 2.0 and 2.3, respectively. Conversely, the Nd: YAG (1064 nm) laser was reported to be less tolerable, with an average pain score of 3.5 (Table 2 and Figs. 1, 2, 3, and 4).

**Table 2:** Average pain score for each laser system based on subject questionnaires

Laser systems	Average Pain Score
Nd: YAG (1064 nm)	3.5
Diode (808–10 nm)	2.0
Alexandrite (755 nm)	2.3

## Discussion

Laser hair removal aims to interrupt the hair regrowth cycle by intervening during the anagen phase, when the hair is most sensitive to treatment, and inducing a kenogen-like phase [7, 8]. Lasers used for medical hair removal include Nd: YAG (1064 nm), diode (808–810 nm), and Alexandrite (755 nm) lasers; each of these lasers is specifically effective for different types of terminal hair (e.g., black, brown, blond hair) [8].

This study introduces a novel hair removal protocol that employs a rotational regimen with three different lasers to enhance treatment efficacy and safety. Before initiating treatment, a thorough preliminary medical examination is essential. During the first visit, detailed biographical data are collected, including the patient’s full name, date of birth, gender, ethnicity, and previous medical procedures. A detailed medical history is reviewed, and an objective examination of the skin and hair in the treatment area is performed. Informed consent is then obtained, ensuring the patient understands the procedure, benefits, risks, and post-treatment care. If necessary, blood chemistry tests are conducted to diagnose conditions like hypertrichosis or hirsutism. Identifying these conditions is crucial because they may indicate underlying hormonal imbalances or other medical issues that could affect the safety and effectiveness of laser treatments. For instance, female patients with hypertrichosis or hirsutism may be suffering from polycystic ovarian syndrome, requiring a different treatment approach or additional medical management to achieve optimal results [7]. This comprehensive approach ensures the treatment is tailored to the patient’s needs and conducted safely.

Once the examination is complete, the medical hair removal treatment, consisting of several continuous sessions, is scheduled. Generally, the treatment is performed once a month for the first three sessions, ensuring all hairs in the targeted area are treated during their anagen phase. After the initial three sessions, hair growth cycles in the treated area become synchronized. The rationale is to progressively target hairs with different characteristics (thickness, color, depth); therefore, subsequent sessions are performed based on hair regrowth until the desired outcome of fine, miniaturized vellus hair is achieved.

Specific lasers are used for different types of terminal hair to maximize treatment efficacy. For dark hair (black to dark brown), the treatment begins with the Nd: YAG laser (1064 nm), which penetrates deeply to effectively target the dense pigment. As the hair becomes thinner and lighter brown after a few sessions, the diode laser (808–810 nm) is used to further reduce hair thickness and pigment. This progression transforms the hair to a finer, blonder state. Finally, the Alexandrite laser (755 nm) is employed due to its optimal wavelength for selective photothermolysis of blonde hair, ultimately achieving the desired hair fleece (kenogen phase), where hair is in its most reduced and least visible form. This sequential use of lasers ensures comprehensive and lasting hair removal by adapting to the changing characteristics of the hair throughout the treatment process.

Medical hair removal laser therapy is tailored to the patient’s phototype and hair color. For patients with skin types IV, V, VI and dark hair, treatment begins with the Nd: YAG laser. For patients with skin types I and II, treatment may start with the Alexandrite laser, which is specific for light hair. All laser systems can be effectively used on fair, tanned, or dark skin, depending on the skin type and using dedicated lasers, such as Soprano in-motion lasers or self-setting lasers like the Vectus diode or Nd: YAG laser [6, 9–11].

The authors’ proposed rotational approach demonstrated significant efficacy, with a 75.07% hair reduction at six months. These results contrast with those obtained in Rao et al.’s prospective study, which evaluated the results of the three different lasers used individually and with a different rotational treatment [12–14]. Their combined treatment, which involved a single session with each of the three lasers, was less effective than the exclusive use of the Alexandrite laser or the 810 nm diode laser, with 31.9 ± 11.1%, 59.3 ± 9.7%, and 58.7 ± 7.7% hair reduction, respectively [12]. The discrepancy in results can be attributed to the different protocols used. In this study, multiple sessions with a single type of laser are conducted until a change in hair type is achieved, making it the optimal target for the subsequent laser. Additionally, a study involving Turkish women with Fitzpatrick skin types II to IV found that after three sessions, hair reduction was 53% for Alexandrite, 50% for IPL, and 39% for Nd: YAG laser systems, which further decreased after six months [15]. Compared to the rotational approach, which maintained high efficacy, the single-laser treatments showed significantly lower long-term hair reduction, emphasizing the benefits of the sequential use of lasers tailored to the evolving characteristics of hair during the treatment process [15].

All subjects tolerated the treatment well, experiencing only a few transient side effects, such as erythema and perifollicular edema. These side effects resolved within a few days and did not result in any long-term complications or adverse effects, as confirmed during follow-up visits. Patient feedback collected through subjective questionnaires indicated an average pain score of 2/4, suggesting mild discomfort during the procedure. This level of discomfort was consistent across different sessions, and no significant variation in pain perception was noted between the various laser types used in the rotational regimen. Overall, the treatment’s tolerability and safety profile were highly favorable, contributing to patient satisfaction and confidence in the procedure’s efficacy. Moreover, no cases of paradoxical hair growth after medical laser treatment [16, 17] have been reported in our patients’ population.

The safety and efficacy of laser therapy in hair removal have been demonstrated over the past 20 years by numerous studies. However, most research has focused on using a single type of laser on different body areas or comparing various laser techniques employed individually or in combination [18–21]. These studies have consistently shown that laser technology not only achieves effective hair reduction but also offers a greater safety profile compared to treatments such as intense pulsed light (IPL). Non-medical lasers and high-intensity IPL devices have been associated with critical safety concerns. For instance, some reports have indicated an increase in “p53” protein levels in IPL-treated patients, which is a known cancer risk factor [22–26]. Consequently, IPL was excluded from the proposed hair removal protocol in this study due to these safety concerns.

This study presents a novel approach by utilizing a rotational regimen combining Nd: YAG, diode, and Alexandrite lasers, tailored to the patient’s skin type and hair characteristics. While this method shows promise in improving treatment outcomes and patient satisfaction, it is important to note several limitations. One is the lack of a control group, which would provide a more rigorous comparison and strengthen the validity of the findings. Despite this, the multicenter retrospective analysis offers valuable insights into the effectiveness and safety of this new laser hair removal protocol. Further research, including randomized controlled trials, is needed to confirm these preliminary results and establish standardized guidelines for optimal hair removal practices. Another limitation is the relatively limited sample size of 60 patients, which may be insufficient for generalizing the findings, and the limited diversity in participants’ age, gender, ethnicity, and skin types restricts the applicability of the results. The six-month follow-up duration may be insufficient for evaluating long-term efficacy and safety; future studies should consider extending the follow-up period to obtain more comprehensive data. The open-label design introduces potential bias because both the researchers and participants are aware of the treatment being administered, which can influence their perceptions and behaviors. For instance, researchers might unconsciously rate the efficacy of the treatment more favorably because they expect positive results. Similarly, participants might report better outcomes or less discomfort because they know they are receiving advanced treatment, a phenomenon known as the placebo effect [27–33]. The subjective nature of pain assessment through questionnaires further compounds this issue, as participants’ self-reported pain levels can be influenced by their expectations and interactions with the researchers. For example, a participant might downplay their discomfort to align with the anticipated success of the treatment. The exclusion criteria limit the generalizability of the findings, as they do not account for patients with certain dermatological conditions or those using specific medications. Moreover, potential variability in image quality and assessment techniques could affect the accuracy of the results. Additionally, inconsistencies in the timing and number of sessions in the rotational regimen protocol might influence its effectiveness. Standardized protocols and further research are needed to confirm these preliminary results and ensure consistent outcomes [4, 21, 34, 35].

## Conclusion

Medical hair removal treatment, utilizing a combination of three different laser technologies (Nd laser, diode laser, and Alexandrite laser), tailored to the patient’s skin type and terminal hair type, promises to be effective, safe, and satisfactory for patients. This approach ensures comprehensive targeting of various hair types and colors, enhancing the treatment’s efficacy. In our hand, the rotational regimen, involving multiple sessions with each laser until optimal hair type transformation is achieved, has shown significant hair reduction with minimal transient side effects and no long-term adverse effects. Anyway, this work is intended as a clinical experience report; therefore, further and more structured studies are needed to confirm the outcomes obtained and to standardize this approach.

## Data Availability

No datasets were generated or analysed during the current study.
